# Efficient nitrogen incorporation in ZnO nanowires

**DOI:** 10.1038/srep13406

**Published:** 2015-08-24

**Authors:** Jan E. Stehr, Weimin M. Chen, Nandanapalli Koteeswara Reddy, Charles W. Tu, Irina A. Buyanova

**Affiliations:** 1 Linköping University, Department of Physics, Chemistry and Biology, Linköping, 581 83, Sweden; 2 Humboldt University, Institute of Chemistry, Berlin, 12489, Germany; 3 University of California, Department of Electrical and Computer Engineering, La Jolla, CA 92093, USA

## Abstract

One-dimensional ZnO nanowires (NWs) are a promising materials system for a variety of applications. Utilization of ZnO, however, requires a good understanding and control of material properties that are largely affected by intrinsic defects and contaminants. In this work we provide experimental evidence for unintentional incorporation of nitrogen in ZnO NWs grown by rapid thermal chemical vapor deposition, from electron paramagnetic resonance spectroscopy. The incorporated nitrogen atoms are concluded to mainly reside at oxygen sites (N_O_). The N_O_ centers are suggested to be located in proximity to the NW surface, based on their reduced optical ionization energy as compared with that in bulk. This implies a lower defect formation energy at the NW surface as compared with its bulk value, consistent with theoretical predictions. The revealed facilitated incorporation of nitrogen in ZnO nanostructures may be advantageous for realizing p-type conducting ZnO via N doping. The awareness of this process can also help to prevent such unintentional doping in structures with desired n-type conductivity.

ZnO has a wide and direct band gap (~3.3 eV at room temperature) and a large exciton binding energy (60 meV at room temperature). It can be easily synthesized in both bulk, single-crystal form and also in a diverse group of nanostructure morphologies. In addition, this material is nontoxic, sustainable and cheap. These attributes make ZnO a very promising material for a wide variety of applications including photonics, electronics, sensing and energy harvesting^1^,^2^,^3^,^4^,^5^. In particular, one-dimensional ZnO nanowires (NWs) have recently attracted a substantial scientific and technological interest for realization of gas sensors and ultraviolet (UV) optoelectronic devices, such as light emitting diodes (LEDs), solar cells, and photo detectors^5^,^6^,^7^,^8^. All these applications, however, require a good understanding and precise control of optical and electrical properties of the material that are known to be largely affected by intrinsic defects and impurities. Zinc vacancies (V_Zn_) for instance, which have the lowest formation energy among all intrinsic defects in n-type ZnO^9^,^10^,^11^, can form detrimental complex centers with group III donors (e.g. Al), thus limiting the n-type doping efficiency^12^,^13^,^14^. On the other hand, they can also facilitate energy upconversion in ZnO^15^,^16^, attractive for photovoltaic applications. Therefore, depending on the intended outcome, defects can be vital or fatal^17^,^18^.

Another group of defects that are important for understanding electrical properties of ZnO involve defects containing nitrogen atoms. Nitrogen has been long considered as one of the most promising candidates for p-type doping in ZnO. Interestingly, even though it has already been proven that nitrogen substituting for oxygen (N_O_)^19^,^20^,^21^,^22^,^23^ does not create a shallow acceptor state, p-type ZnO using nitrogen as a dopant has been commonly reported^24^,^25^,^26^. So far, it is still under discussion how nitrogen doping leads to the formation of shallow acceptors in ZnO. Here one needs to keep in mind that the doping processes can be largely affected not only by growth conditions and type/concentration of the utilized dopants, but also residual contamination of the source material or even the background gases in the growth chamber^27^. For example, it was reported that p-type conductivity in ZnO could be promoted in nanostructured materials^25^,^28^,^29^. Though the exact physical mechanism responsible for this effect remains unknown, it could be caused by formation of complexes with residual defect/impurities. Indeed, the energy level of the N acceptor can be affected by its local surrounding^30^, complexing with residual group III impurities^31^ and defects^32^,^33^, as well as formation of N-N molecules^34^. Moreover, growth conditions utilized during the NW growth may facilitate dopant incorporation leading to higher dopability of these materials. In this letter we employ magnetic resonance spectroscopy^35^ to investigate defect formation processes in nominally undoped ZnO NWs grown by chemical vapor deposition, aiming to single out chemical origin of incorporated contaminants.

## Results and Discussion

**Figure 1:**
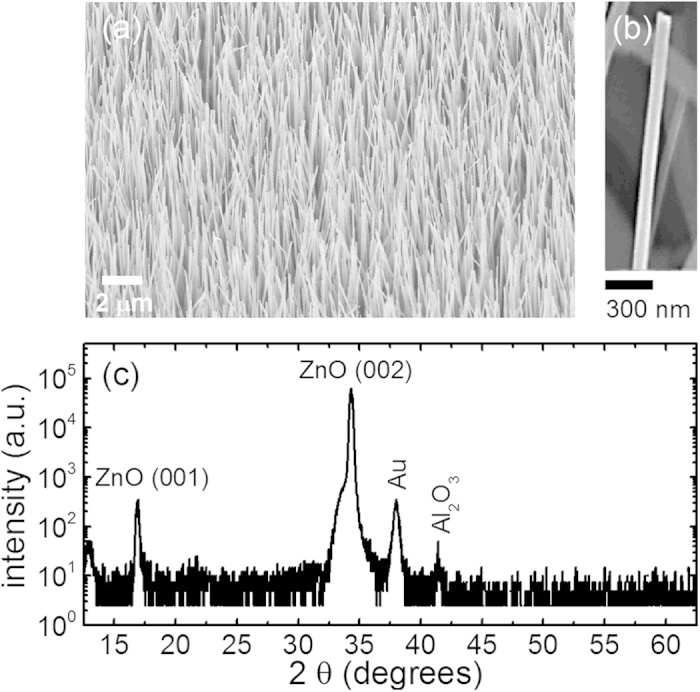
(**a**) An SEM overview image of the studied ZnO NWs under a tilted view of 45°. (**b**) a magnified SEM image of a single ZnO NW. (**c**) XRD diffractogram of the studied ZnO NWs.

Figure 1(a) shows a representative scanning electron microscopy (SEM) image of the investigated ZnO NWs. A magnified image from a single NW is shown in Fig. 1(b). Most of the NWs are found to be vertically aligned along the crystallographic [0001] axis and exhibit a uniform size distribution with an average length and diameter of 30 μm and 100 nm, respectively. Some of the NWs are, however, randomly tilted by up to 20°. The excellent structural quality was further confirmed from performed x-ray diffraction (XRD) experiments, The XRD spectra of the ZnO NWs contain four peaks which can be assigned to ZnO (001) and (002) reflexes, Au and Al_2_O_3_ - see Fig. 1(c). The latter two reflexes are expected since the NWs were grown on an Al_2_O_3_ substrate and Au was used as a catalyst. The XRD results confirm that the NWs are preferentially oriented along the crystallographic c-axis ([0001] direction), since the calculated d-spacing of the major reflex (2θ = 34.4°, d = 0.261 nm) matches that expected for hexagonal ZnO. The full width at half maximum (FWHM) of the main ZnO reflex was found to be 0.27°, indicative of the high quality ZnO.

**Figure 2:**
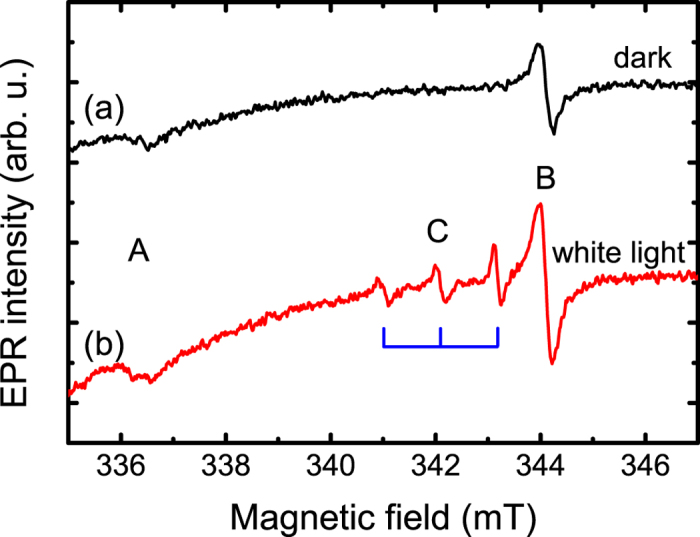
EPR spectra of the studied ZnO NWs measured at 4 K and a microwave frequency of 9.4 GHz in the dark (**a**) and under white light illumination (**b**) with an applied magnetic field rotated away from the NW growth axis ([0001], i.e. the c - axis) by Θ = 70°.

Figure 2 depicts EPR spectra of the ZnO NW arrays measured in dark (a) and under white light illumination (b). In the dark, two single-line EPR signals labeled as A and B in Fig. 2 can be observed. White light illumination leads to appearance of another EPR signal (labeled as C) that consists of three equally spaced lines, which is characteristic for a resolved hyperfine interaction involving a nucleus spin I = 1 with natural abundance of 100%.

**Figure 3:**
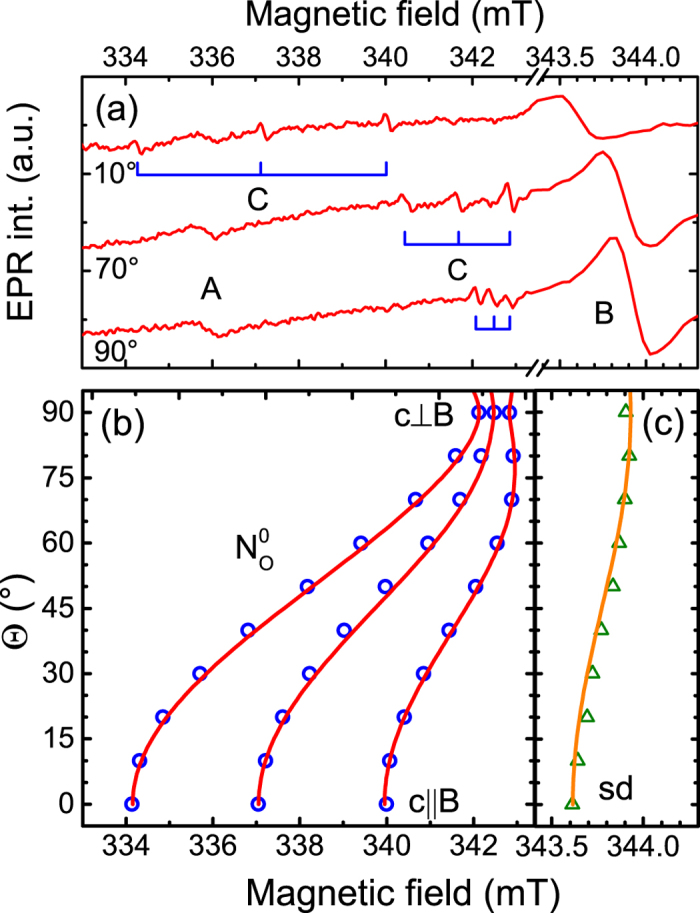
(**a**) EPR spectra of the ZnO NWs measured at 4 K under white light illumination with an applied magnetic field rotated away from the NW growth axis ([0001], i.e. the c - axis) by Θ = 10°, 70° and 90°. (**b**) Angular dependent plot of the N_O_ EPR field positions (the open circles) and the best fit using the spin-Hamiltonian described in the text (the solid lines). (**c**) Anisotropic behavior of signal B, i.e. the shallow donor (sd) EPR field postions (the open triangles) obtained by rotating a magnetic field direction from B parallel to c to B perpendicular to c. The solid line is the best fit obtained by using the spin-Hamiltonian described in the text. (Note different x-axis scales in (**b**,**c**)).

In order to identify the observed EPR signals we performed angular dependent measurements under light illumination by rotating the sample from an orientation with the NWs aligned parallel to a static magnetic field (**B**), i.e. Θ = 0°, towards Θ = 90°. In the latter case **B** is oriented perpendicularly to the [0001] NWs axis. Figure 3(a) shows representative results from these measurements, taking as an example EPR spectra measured with Θ of 10°, 70° and 90°. An anisotropic behavior for signals B and C can be clearly observed, while signal A is isotropic. By using a spin-Hamiltonian operator in the form of


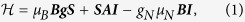


i.e. including the Zeeman energy (the first term), the hyperfine interaction (the second term) and the nuclear Zeeman interaction (the third term), we can determine spin-Hamiltonian parameters of the observed EPR signals. Here we neglect the quadrupole interaction for nuclei with I > 1/2, since the EPR signal intensity of the forbidden transitions arising from the quadrupole interaction is too weak to determine its interaction tensor. It is found that signal A has an electron spin S = 1/2 and an isotropic g-value of 2.002. Within the experimental error, this value is identical to the free electron g-value g_e_ = 2.0023. We, therefore, assign this signal to dangling bonds on the surface of the NWs^36^,^37^, which seems to be very likely considering a large surface-to-volume ratio in the NWs. Signal B has an electron spin S = 1/2 and a slightly anisotropic g-tensor with g_||_ = 1.957 and g_^_ = 1.955 (see Fig. 3(c)), where parallel and perpendicular orientations are given with respect to the crystallographic c-axis. Such g-values are typical for shallow effective mass donors in ZnO^35^. Judging from the determined g-values, this shallow donor is most likely caused by unintentionally incorporated hydrogen^38^. Unfortunately the corresponding hyperfine interaction (1.4 MHz) is too small to be resolved in the X-band EPR experiments conducted in this work.

Let us now discuss the chemical origin of signal C. First of all we note that it must arise from a chemical element that has an isotope with a nuclear spin I = 1 and nearly 100% natural abundance. The only element in the periodic table which satisfies this requirement is nitrogen as the ^14^N isotope has I = 1 and 99.6% natural abundance. Moreover, signal C exhibits strongly anisotropic behavior with an angular dependent g-value and the hyperfine interaction parameter A. This can also be seen from Fig. 3(b) where the dependence of signal C on the orientation of the external magnetic field is depicted. By fitting the experimental data with the spin-Hamiltonian given by Eq. (1) we obtain g_||_ = 1.996, g_^_ = 1.963, A_||_ = 82.3 MHz and A_^_ = 9.7 MHz. The determined spin-Hamiltonian parameters are very similar to those reported in the literature for the substitutional N_O_ center in bulk ZnO^20^,^39^. We therefore identify signal C as arising from a nitrogen atom substituting oxygen. In bulk, a typical linewidth ΔB of the N_O_ EPR lines is about 0.03 mT and slightly deviates between the lines, which can be explained by inhomogeneity of the g-values and hyperfine interaction^39^. An additional broadening (up to 0.2 mT) of the C lines in the studied NWs is likely caused by imperfect perpendicular alignments of the NWs relative to the substrate surface, evident from their random tilt directions and angles up to 20° from the vertical direction as revealed from the performed scanning electron microscopy measurements^40^.

**Figure 4:**
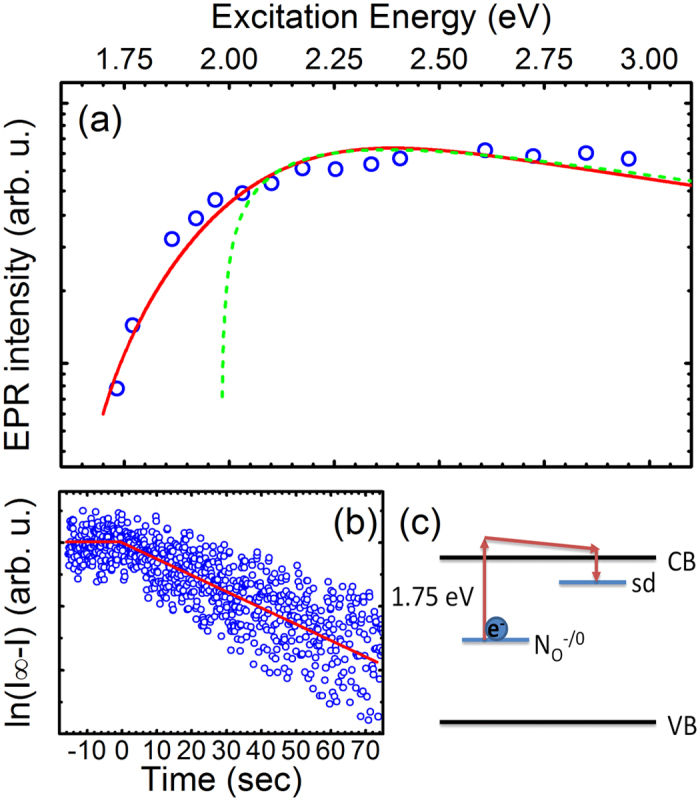
(**a**) EPR intensity of the N_O_ signal (open circles) as a function of the light excitation energy for the studied ZnO NWs and bulk ZnO (dashed line). The solid line is a guide to the eye. (**b**) Time dependent behavior of the N_O_ signal after switching on the light. The y-axis displays the difference of the measured EPR intensity (I) from the saturation value (I_∞_) shown in a logarithmic scale. The linear slope shown by the solid line indicates a mono-exponential process due to direct recharging. (**c**) Illustration of the N_O_ photo-EPR recharging process. The notation “sd” denotes the shallow donor center.

The energy level position of the N_O_ center in the ZnO NWs can be determined from photo-EPR measurements. The measured spectral dependence of the N_O_ EPR signal on the excitation photon energy is shown by the open circles in Fig. 4(a). We can see that the N_O_ center can be converted into its paramagnetic state 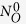
only when the photon energies exceed 1.75 eV. The recharging process can be further understood by analyzing temporal behavior of the N_O_ signal after switching on the light. The corresponding results are shown in Fig. 4(b), where the y-axis displays the difference of the measured EPR intensity (I) from the saturation value (I_∞_) shown in a logarithmic scale. The linear slope shown by the solid line indicates a mono-exponential process due to direct recharging of the studied center without involvement of other defects. Moreover, since light illumination within the same spectral range also causes an increase of the shallow donor EPR signal (see Fig. 2), the recharging process results in photo-ionization involving the conduction band. This means that the light-induced conversion of the N_O_ center into its paramagnetic charge state occurs as a result of the process


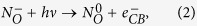


as schematically shown in Fig. 4(c).

It is interesting to note that the photo-ionization threshold energy in the NWs differs from that in bulk ZnO where the threshold energy is found to be at around 2 eV (see the dashed curve in Fig. 4(a)), which is in agreement with previous reports^20^. One possible explanation for this deviation is that, unlike in bulk ZnO, the N_O_ centers in the NWs are not distributed uniformly through the volume but are located in proximity to the NW surface. (The possibility of the N_O_ centers to be directly located on the NW surface can be excluded, however, since this would drastically alter the spin-Hamiltonian parameters of the centers, which is not the case). In fact previous theoretical studies^41^,^42^ have concluded that the ionization energies of donor and acceptor impurities are strongly enhanced in nanowires with respect to their values in bulk. Moreover, due to surface band bending^43^ optical ionization of the 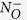
center within the near-surface region can occur not only due to spatially direct, but also spatially indirect optical transitions. In addition, the electron-phonon interactions for the 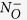
center, which significantly affect the photoionization edge, could be altered in the proximity to the surface. Both effects will lead to an overall broadening of the ionization edge, as is observed experimentally. This suggestion is also consistent with the theoretical results of Gutjahr *et al.*^44^ and Haffad *et al.*^45^, which predicted that incorporation of nitrogen in ZnO is more energetically favorable at (or close to) the surface than in volume regions. This is expected to result in a higher efficiency of N doping in nanostructures with a high surface-to-volume ratio. Gutjahr *et al.*^44^ have explained the decrease in the formation energy by charge transfer between Zn dangling bonds on the surface and the N_O_ impurities. Existence of such dangling bonds on the surface of the studied NWs is indeed confirmed by the observation of signal A in our EPR measurements.

Of course the formation energy of N_O_ in bulk and nanostructured ZnO is dependent on the growth conditions (O-rich or Zn-rich) and the Fermi level position, which is strongly affected by the dopant concentration. However, these dependencies are known from ab initio density functional theory (DFT) calculations^22^,^23^. For examples, the formation energy scales linearly with the Fermi level position (determined by the dopant concentration) under fixed growth conditions (Zn- or O-rich), since effects of lattice relaxation at doping concentration lower than 6.06% are negligible^45^. Therefore, our conclusion of the reduced formation energy of the N_O_ centers in the NWs, though obtained for low-doped ZnO, should remain valid even for higher doping concentrations.

**Figure 5:**
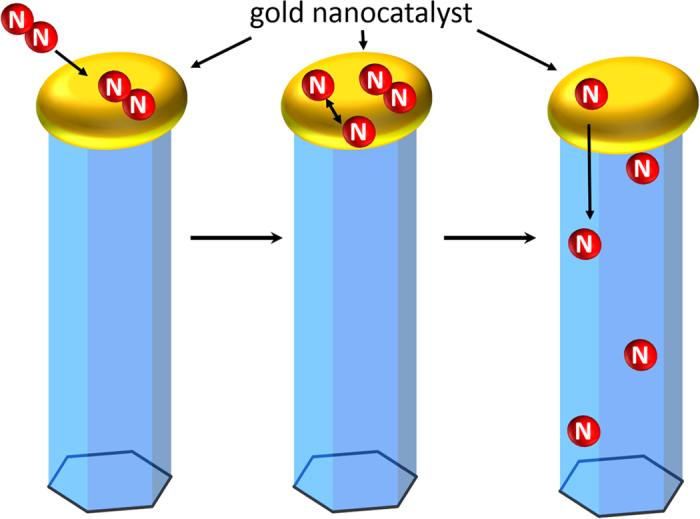
Schematic illustration of the nitrogen incorporation into the ZnO NWs from the residual background gases in the growth chamber that contain N_2_.

The remaining and somewhat puzzling questions are what are the sources and mechanism responsible for the revealed unintentional doping of the ZnO NWs by nitrogen. The studied structures were grown by chemical vapor deposition in an Ar ambient using gold as a catalyst. Therefore, nitrogen atoms were most likely supplied by contaminations in the used Ar_2_ and O_2_ source gases and/or by residual background N_2_ gas. Both source gases had a purity of 6N (99.9999%) and hence can contain up to 1 ppm N_2_. The contamination of the residual background gas most likely stems from venting the growth chamber with N_2_ gas after the deposition of the gold catalyst on the sapphire substrate prior to the growth of the NWs. We believe that the N_2_ contamination from the used Ar_2_ and O_2_ source gases dominates, since defect incorporation due to contaminations of the used source gases is a known phenomenon in RTCVD processes^46^,^47^ and was shown to prevail over contamination by residual chamber background gases^46^. According to previous studies^27^,^48^, nitrogen molecules can be dissolved easily in molten metals and subsequently split into atomic nitrogen. This process happens when metal is heated to high temperatures (T_Growth_ = 950 °C) under the presence of molecular nitrogen gas. Under these conditions nitrogen molecules permeate into the metal and nitridation proceeds by dissociation of nitrogen molecules and absorption of nitrogen atoms by the metal^48^. Taking into account the large surface area of the gold catalyst during the growth, this process can be rather efficient. The dissolved atomic nitrogen could then incorporate and diffuse (comment: diffusion may happen but is not necessary, as the Au droplet is floating on the surface of the growing NWs) into the growing NWs and predominantly reside at oxygen sites giving rise to the N_O_ EPR signal. An illustration of the suggested process of nitrogen incorporation into the ZnO NWs is shown in Fig. 5. We would like to note that considering a limited amount of nitrogen in the growth chamber (only a few ppm), the dopant incorporation efficiency during the revealed doping process must be very high. Indeed, the concentration of the N_O_ centers estimated based on the EPR signal intensity is about 2–4 × 10^16^ cm^−3^, which is only by about one order of magnitude lower than the deduced concentration of the residual shallow donors (signal B) of 2–4 × 10^17^ cm^−3^ in the NWs. The low formation energy of the substitutional nitrogen center in ZnO nanostructures is advantageous for the synthesis of p-type conductive via nitrogen doping, e.g. due to N-containing defect complexes or nitrogen molecules. The awareness of this process can also help to prevent such unintentional doping in structures where n-type conductivity is desired, as N_O_ can act as a compensation center in n-type ZnO. Unfortunately, it is not possible to directly compare the efficiency of the N_O_ incorporation during the employed growth with that achieved previously in intentionally N-doped ZnO NWs, since the information on the N concentration in the wires was not provided in most of the earlier reports^27^,^49^,^50^. Yuan *et al.*^24^ estimated that the carrier concentration in their N-doped ZnO NWs grown by chemical vapor deposition (CVD) is ~1 × 10^18^ cm^−3^. However, the exact configuration of the incorporated nitrogen (N_O_, N-molecules or N-complexes) and its concentration were not determined.

## Conclusion

In summary, we have provided an unambiguous experimental proof for efficient unintentional doping with nitrogen in ZnO NWs during the RTCVD growth process. Based on our results from the detailed EPR measurements, incorporated nitrogen is shown to reside at oxygen sites forming a substitutional N_O_ acceptor. Based on the lower photo-ionization threshold energy of the N_O_ center in the ZnO NWs as compared with its value in bulk ZnO, the defect is suggested to be located in proximity to the surface. This assumption is consistent with theoretical predictions^44^,^45^ of enhanced N incorporation at or close to the ZnO surface. The revealed doping process is shown to be very efficient leading to a rather high concentration (~2–4 × 10^16^ cm^−3^) of unintentional N dopants in the NWs even from the contamination by the source and background gases. Our finding thus underlines the importance of controlling such background contamination as compensation by the N_O_ acceptors may hinder achieving high n-type conductivity in nanostructured ZnO. On the other hand, the lowered formation energy of N_O_ in NWs might be beneficial for achieving p-type conducting ZnO nanostructures via nitrogen doping, e.g. due to N-containing defect complexes or nitrogen molecules.

## Methods

The ZnO NWs were grown on gold coated c-plane sapphire substrates by using rapid thermal chemical vapor deposition (RTCVD). The growth was performed at a growth temperature of 950 °C under pressure of 20 Torr with Ar_2_ and O_2_ flows of 100 and 2 sccm, respectively^40^. The Au catalyst had a thickness of about 3 nm and was deposited using e-beam evaporation. Most of the NWs are vertically aligned along the crystallographic [0001] axis and exhibit a uniform size distribution with an average length and diameter of 30 μm and 100 nm, respectively. Some of the NWs are, however, randomly tilted by up to 20°.

EPR experiments were performed at a microwave frequency of 9.4 GHz (X-band) at a temperature of 4.2 K. To perform photo-EPR measurements, a Xenon lamp was used as an excitation source. Appropriate long-pass and short-pass filters were utilized to select specific illumination wavelengths whereas neutral density filters were inserted to ensure the same excitation power at all chosen wavelengths. To avoid effects of ambient illumination and to guarantee the same initial conditions, the sample was cooled down each time in the dark before illumination. Time-dependent photo-EPR measurements were performed at a constant magnetic field corresponding to the EPR peak position and the EPR signal strength was monitored as a function of time after switching on the light.

## Additional Information

**How to cite this article**: Stehr, J. E. *et al.* Efficient nitrogen incorporation in ZnO nanowires. *Sci. Rep.* **5**, 13406; doi: 10.1038/srep13406 (2015).
